# Consistency in phonetic categorization predicts successful speech-in-noise perception

**DOI:** 10.1121/10.0041846

**Published:** 2025-12-10

**Authors:** Rose Rizzi, Gavin M. Bidelman

**Affiliations:** 1 Department of Speech, Language and Hearing Sciences, Indiana University, Bloomington, Indiana 47408, USA; 2 Program in Neuroscience, Indiana University, 47405, USA; 3 Cognitive Science Program, Indiana University, 47405, USA

## Abstract

Listeners bin continuous changes in the speech signal into phonetic categories but vary in how consistently/discretely they do so. Categorization may relate to speech-in-noise (SIN) perception. Yet, it is unclear if and how perceptual gradience, consistency, and other cognitive factors (e.g., working memory) collectively predict SIN performance. Here, we estimated perceptual gradiency and response consistency during vowel labeling and assessed working memory and SIN performance. We found perceptual consistency and working memory were the best predictors of listeners' SIN scores. Our findings emphasize the importance of perceptual consistency over categoricity for noise-degraded speech perception.

## 1. Introduction

To deal with continuous variance in the speech signal, listeners bin speech sounds into phonetic categories.^1,2^ The classic view of categorization suggests that listeners use a small number of auditory cues to inform category membership and discard irrelevant, within-category acoustic information.^3,4^ Yet, studies show listeners have access to both between- and within-category information^5,6^ and vary in how they weight this information. Some listeners have a more continuous perceptual representation of the speech signal (gradient listener), whereas others strongly warp the acoustics onto distinct phonetic categories (discrete listener).^7,8^ A person's listening strategy, (i.e., degree of perceptual gradiency), can be measured *via* the slope of their identification curves derived from labeling tokens along an acoustic-phonetic continuum. More gradient listeners have shallower identification,^7^ consistent with weaker categorical percepts and more continuous hearing.

Individual differences in categorization are not restricted only to perceptual gradiency. Listeners also vary in how *consistently* they label tokens along the continuum.^7,9,10^ Consistency may be a more precise measure of individual differences in categorization than slope, accounting for intertrial differences in perceptual reports.^11^ Whether consistency and gradiency are independent measures remains debated,^7,8,10,12^ but measuring both slope (gradiency) and intertrial variance (consistency) allows them to be directly compared and considered as covarying factors in speech perception.^13^

Gradiency in phoneme categorization has been linked to other perceptual processes as well, including increased perceptual flexibility,^7,14^ linguistic diversity,^10^ and indexical variation.^15^ On the other hand, poorer consistency is linked to speech-language problems including developmental language disorder and dyslexia,^16^ emphasizing the importance of stable perceptual sound representations to broader auditory-linguistic skills. More consistent listeners also seem to have an advantage for second language learning.^17,18^

**Fig. 1.**
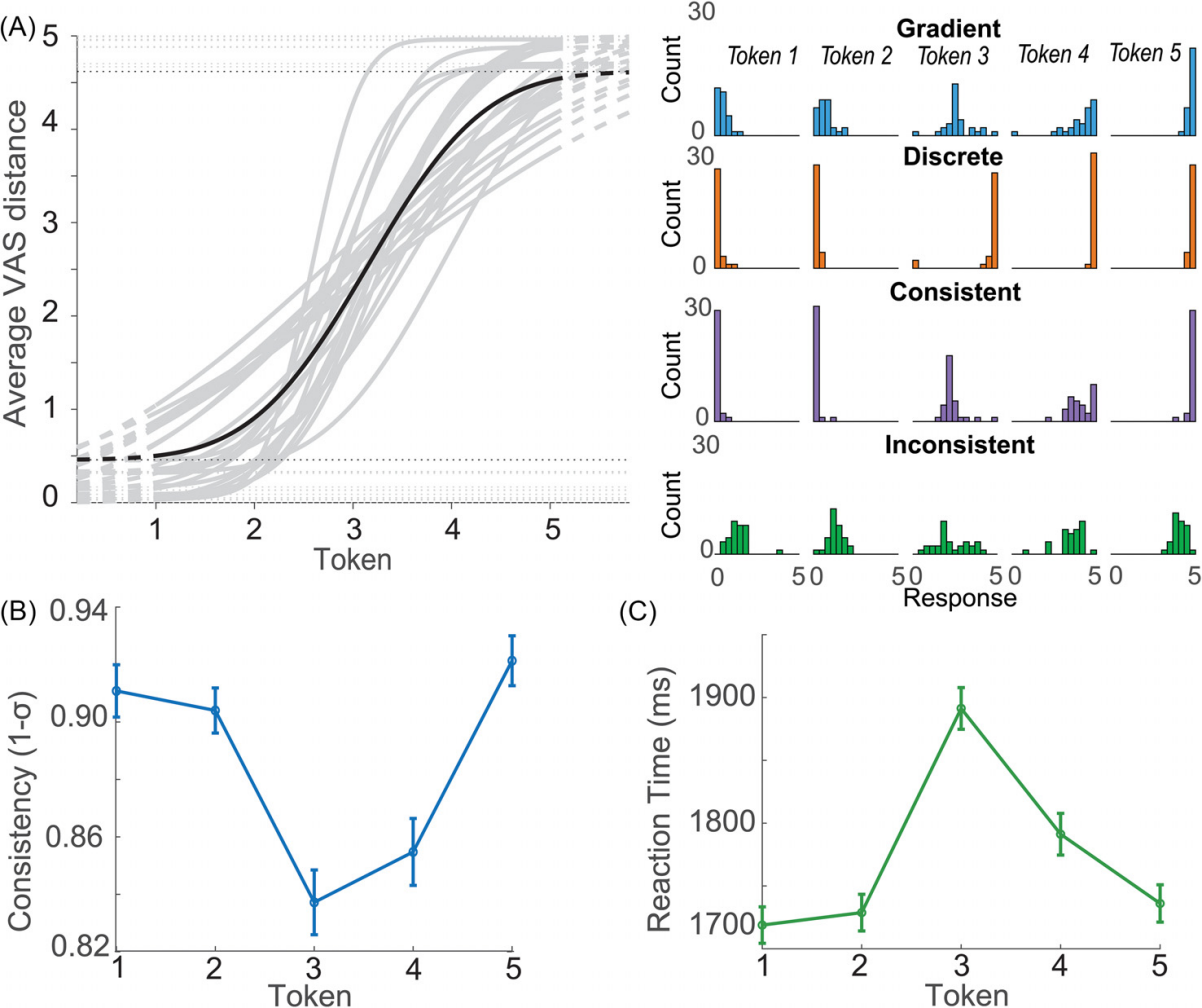
Listeners vary in categorization behavior. (A) Left, phoneme identification curves from each participant (gray traces); grand average (black bold). Right, VAS response distributions across continuum steps for representative subjects with different gradiency/consistency response patterns. (B) Consistency (1–σ) across tokens. Listeners' labeling is less consistent and more variable in the middle of the continuum. (**C**) RTs for phoneme labeling. Both consistency and RTs are poorer near the continuum midpoint (Tk 3). Error bars = ±s.e.m.

Classic categorization experiments have used two-alternative forced choice (2AFC) paradigms, requiring listeners to make a binary decision. Consequently, categories might be an artifact of task demands rather than a true perceptual phenomenon.^15,19^ The 2AFC tasks confound whether a shallower identification slope represents a more gradient listener or a less consistent labeler.^7,20^ Recent work favors a visual analog scale (VAS) to assess categorization, which allows for more gradiency in perceptual reports.^7,20–22^ Both the slope of the identification curve and intertrial consistency can be quantified and disentangled from VAS responses.^7,20^ For instance, discrete listeners will primarily use the end points of the scale to make their reports, but how consistently they choose the same end point for a given token may vary. Gradient listeners will have a distribution of responses that moves down the scale mirroring changes in the acoustic cue, although the spread of token-wise distributions may vary [see Fig. 1(A)].

Having a more consistent internal representation of speech sounds might also allow listeners to better parse speech from noise. Additionally, maintaining perceptual flexibility with gradient hearing may allow better recovery from ambiguity introduced by noise degradation. However, whether categorization skills predict SIN performance is equivocal. Recent work demonstrates both increased consistency and gradience in categorization correspond with better SIN comprehension,^8,12^ but these studies did not control for working memory (WM). Kapnoula *et al.*^7^ found the relationship between slope gradience and SIN comprehension dissolves when controlling for WM, a known cognitive factor important for SIN outcomes.^23–26^ However, these studies only used (i) the QuickSIN to assess SIN performance and (ii) either slope or consistency, so it is unclear whether these effects generalize across different SIN tests and properties of categorization. How perceptual (gradiency, consistency) and cognitive (WM) factors contribute to and potentially interact in SIN performance remains an open question. We directly pitted these factors against one another in the current study to assess the degree to which categorization (gradiency, consistency) and WM explain SIN outcomes.

## 2. Methods and materials

### 2.1 Participants

The sample included *N* = 24 normal hearing (pure-tone thresholds ≤25 dB HL; 250–8000 Hz) adults aged 18–41 (mean = 24.89 ± 6.1 years; 12 female, 12 male). Participants were native speakers of American English and had 9.04 ± 7.0 years of music training. All provided written informed consent in accordance with the Indiana University Institutional Review Board.

### 2.2 Stimuli and tasks

*Phoneme categorization*. Stimuli consisted of five vowel tokens equidistantly sampled from a first formant frequency (F1) continuum changing from/u/(430 Hz) to/ɑ/(730 Hz).^27,28^ Tokens were otherwise identical with respect to F0 (150 Hz), F2 (1090 Hz), and F3 (2350) and were 100 ms in duration (10 ms ramps). Stimuli were generated *via* conventional source-filter (cascade formant filter) synthesis in matlab.^27^ They were presented at 75 dB sound pressure level (SPL) *via* PsychoPy (version 2023.1.1)^29^ through Sennheiser HD 280 circumaural headphones (Sennheiser, Wedemark, Germany). Listeners heard 30 presentations of each vowel (total = 150 trials). The interstimulus interval between trials was 500 ms. On each trial, they clicked along a visual analog scale (VAS) with end points labeled “oo” and “ah” to report what they heard.^7,8^ They were not explicitly instructed to provide speeded responses. Average VAS distance was reported and reaction times (RTs) were scored per token.

*Speech-in-noise (SIN).* We assessed SIN abilities using the QuickSIN,^30^ Hearing in Noise Test (HINT),^31^ and Words in Noise (WIN) test.^32^ SIN tests were administered *via* matlab at 70 dB HL (QuickSIN), 65 dB-A (HINT), and 75 dB SPL (WIN). The QuickSIN uses six low-context sentences with four-talker babble. The signal-to-noise ratio (SNR) decreases from 25 dB to 0 dB in 5 dB steps with each sentence. The HINT uses 20 higher-context sentences with a speech-shaped noise masker and an adaptive SNR starting at 24 dB that tracks down to the listener's threshold (SNR-50). For WIN, monosyllabic words follow a carrier phrase “say the word” with six-talker babble at an SNR that decreases by 4 dB every 5 words from 24 dB to 0 dB. We used 35 words for each list. All three SIN assessments calculate the listener's SNR-50, the SNR at which they can correctly repeat 50% of the keywords. The final QuickSIN score is represented as SNR loss, a measure of how much higher the listener's SNR-50 is relative to clinical norms. Lower scores correspond to higher tolerance for noise and thus better SIN performance. For all tests, performance was averaged across two lists.

*WM.* We assessed auditory WM capacity using the forward and backward digit span.^33^ A series of digits was verbally presented to listeners (∼1/s) which varied in sequence length. The number of items progressively increased from two to nine in the forward condition and two to eight in the backward condition (two trials of each item). Participants scored one point for each sequence correctly recalled. Participants were required to recall the sequence in serial order for the forward condition and reverse order from the presentation for the backward condition. The test was discontinued if the participant missed both trials. WM spans were computed as the number of correct responses of 16 and 14 possible points for forward and backward directions, respectively.

### 2.3 Data analysis

Phoneme identification curves were fit with a sigmoid P = 1/[1 + e^−β1(x−β0)^] and *β1* slopes were estimated using the matlab psignifit toolbox.^34^ Response consistency of labeling was calculated as 1–*σ_Tk_*, where *σ* is the standard deviation of VAS responses to each token (*Tk*) for each participant.^7^ RTs in labeling speed were computed as the trimmed mean response latency for each token per condition; RTs outside 250–3000 ms were deemed lapses of attention or fast guesses and were excluded.^28,35^

We used linear mixed-models in r version 4.5 lme4 package^36^ to analyze the dependent variables. Separate models were built to test differences in consistency and RTs, with a fixed effect for token (five levels; Tk1-5) and random intercepts for subject [e.g., RT ∼ Tk + (1|sub)]. Pairwise comparisons were Tukey-adjusted. Effect sizes were calculated as $\eta_{p}^{2}$. We used linear regressions to assess relationships between SIN scores, WM digit span scores, and phoneme labeling slope and consistency (e.g., SIN test score ∼ consistency + slope + total digit span score + music experience + age). Music experience and age were also included in the model as they are factors known to affect both SIN perception^23,37,38^ and categorization.^39–41^ Separate models were run for each SIN test (i.e., QuickSIN, HINT, WIN scores). Consistency and slope were not allowed to interact in the model to avoid multicollinearity. Variance inflation factors for our model ranged from 1.14 to 2.52 indicating multicollinearity was negligible.^42^ We assessed relationships between WM (digit span scores) and categorization using Pearson's correlations.

## 3. Results

We first confirmed there were individual differences in speech categorization by visualizing identification curves and VAS response distributions. Figure 1(A) shows phoneme labeling functions derived from listeners' VAS distributions. The steepness of the functions varies across listeners, suggesting differences in the degree of continuous vs categorical perception (i.e., listening strategy). Representative discrete, gradient, consistent, and inconsistent VAS response patterns across continuum steps are shown on the right. Categorization gradiency is evident in how the responses track across steps, whereas response consistency is evident in the width of distributions around each token. Gradient listeners parametrically shift their perceptual response along the scale with each continuum step, whereas discrete listeners heavily use end points of the scale to respond. More consistent listeners have a smaller spread in their VAS response distributions than inconsistent listeners. Slope and average consistency were weakly correlated [*r*(22) = 0.42, *p* = 0.044], suggesting these measures are partially separable.

We next assessed the effects of stimulus token on labeling consistency. We found a main effect of stimulus token on labeling consistency [*F*(4,92) = 16.67, *p* < 0.0001, $\eta_{p}^{2}$ = 0.42]. This was driven by less consistent labeling of the midpoint token [all (Tk1,2,5) vs Tk3: *p* < 0.0001, except Tk3-4 contrast: *p* = 0.64] [Fig. 1(B)]. Likewise, RTs showed a main effect of token on labeling speeds [*F*(4,92) = 16.84, *p* < 0.0001, $\eta_{p}^{2}$ = 0.42], driven by slower RTs for Tk3 [(Tk1,2,4,5) vs Tk3: *p* < 0.006] [Fig. 1(C)].

**Fig. 2.**
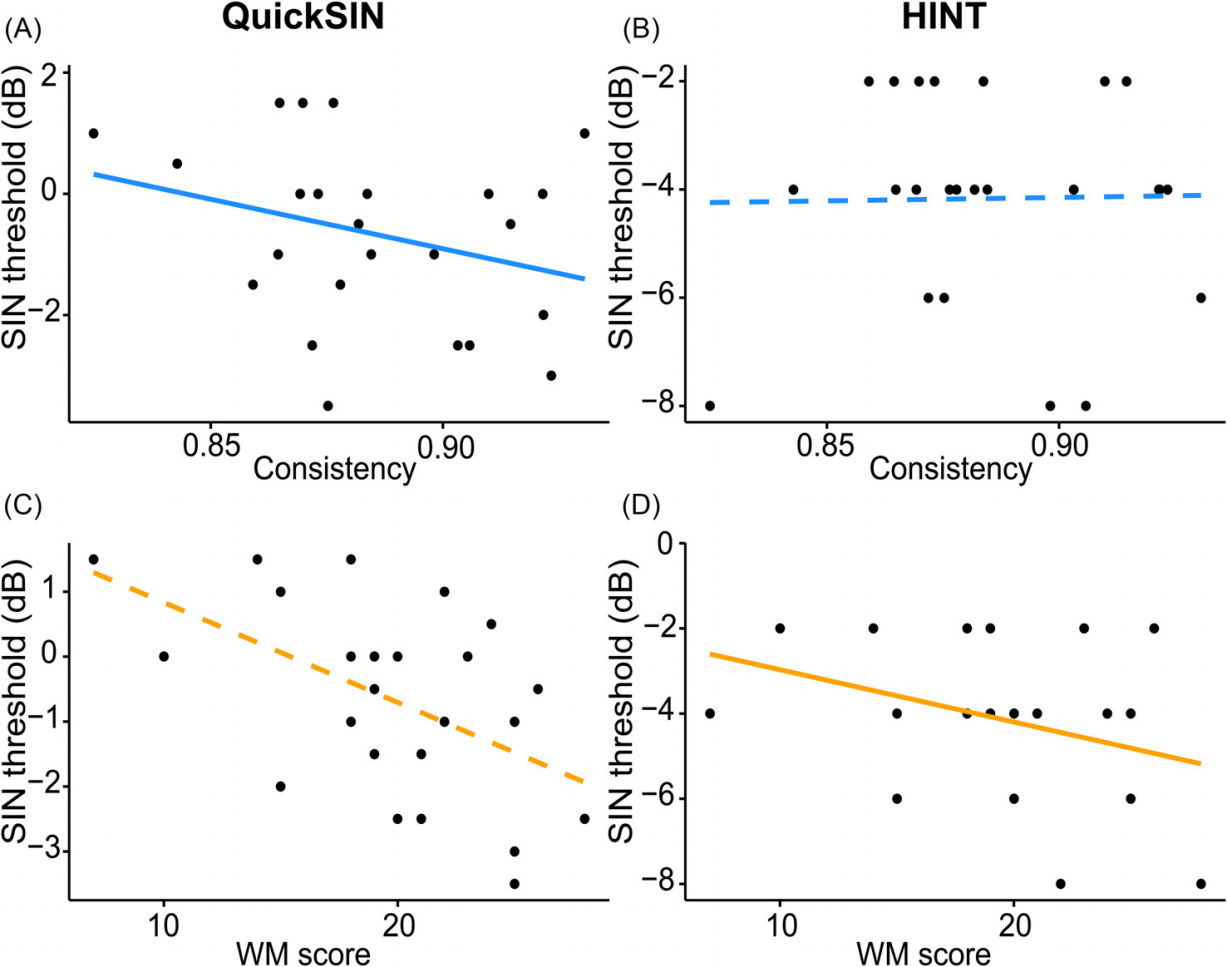
Scatterplots showing relationships between SIN test scores vs phoneme labeling consistency (**A, B**) and total digit span scores (WM) (**C, D**). Left column, QuickSIN scores; right column, HINT scores (nonsignificant WIN data not shown). Solid lines indicate significant predictors from linear regression. Better QuickSIN scores are predicted by more consistent labeling, whereas better HINT scores are predicted by higher WM capacity.

We next assessed whether phoneme labeling consistency, slope, and/or WM digit span scores predicted SIN scores. Forward and reverse digit span scores were not significantly correlated [*r*(22) = 0.38, *p* = 0.064]. We used a composite digit span score (total of forward/backward scores) to measure overall WM (although statistics were similar using only reverse digit span scores, which had greater variability than forward span scores). Linear regression for the QuickSIN showed consistency [*F*(1) = 8.14, *p* = 0.011, $\eta_{p}^{2}$ = 0.31], and age [*F*(1) = 8.12, *p* = 0.011, $\eta_{p}^{2}$ = 0.31] significantly predicted QuickSIN scores, whereas slope [*F*(1) = 2.94, *p* = 0.103, $\eta_{p}^{2}$ = 0.14], total digit span [*F*(1) = 3.13, *p* = 0.094, $\eta_{p}^{2}$ = 0.15], and music training [*F*(1) = 0.14, *p* = 0.716, $\eta_{p}^{2}$ < 0.001] did not [Figs. 2(A) and 2(C)]. HINT scores were predicted only by total digit span scores [*F*(1) = 6.23, *p* = 0.023, $\eta_{p}^{2}$ = 0.26] (all other *p* values > 0.23) [Figs. 2(B) and 2(D)]. WIN scores were not predicted by any of the regressors (all *p* > 0.06). WM scores were correlated with slope [*r*(22) = −0.60, *p* = 0.002] but not consistency [*r*(22) = 0.08, *p* = 0.711].

## 4. Discussion

Prior work has implied a potential link between categorization skills (i.e., acoustic-to-phonetic mapping of phonemes) and noise-degraded speech perception.^7,8,12,43^ Here, we explicitly assessed whether phoneme labeling and WM predicted SIN scores measured in the same listeners. We found perceptual labeling was least consistent for tokens in the middle of the acoustic-phonetic continuum. Phoneme labeling consistency predicted QuickSIN scores, whereas WM capacity predicted HINT scores with greater consistency and WM corresponding with better (lower) SIN scores on respective assessments. These results establish a link between categorization consistency and sentence perception skills in noise controlling for WM abilities.

Labeling consistency and identification curve slope varied across listeners and were only weakly correlated. More discrete listeners (i.e., with steeper slopes) tended to be more consistent labelers. Whether categorization gradiency and consistency in speech perception are independent constructs is debated.^7,8,10,12^ Instead, listeners might fall into at least one of three sub-types of “speech categorizer” in terms of their perceptual gradiency and consistency—canonically gradient, canonically categorical, or categorical but noisy.^10^ Instead of grouping listeners as in prior work, we treated categorization slope and consistency as continuous measures and included both as covarying effects in our analysis. This revealed that consistency in listeners' perceptual labeling was related to their QuickSIN performance, even after controlling for the degree of gradiency/discreteness in their hearing. Although some studies suggest perceptual gradiency is advantageous for SIN recognition,^8,12^ our findings here suggest increased perceptual *consistency* better predicts noise-degraded listening skills.

Although we found categorization consistency predicted QuickSIN performance, it did not predict HINT or WIN scores. Other studies have found similar patterns in listeners with different auditory expertise where large individual differences in SIN perception are restricted to the QuickSIN.^23,44^. This is likely because of differences in the complexity of the SIN assessments. WIN targets are monosyllabic words, inherently less linguistically complex and requiring less cognitive skill for correct recall than sentences. In contrast, the QuickSIN is a sentence-level test that uses more complex materials (i.e., longer and lower-context sentences) than the HINT.^45^ The QuickSIN also uses multi-talker babble (informational masker), whereas the HINT used a speech-shaped noise (more energetic masker). Thus, it is possible the benefits of phoneme consistency on broader SIN perception may be restricted to more demanding materials that draw on higher level aspects of perception and the disentanglement of multiple competing speech signals—as assessed by the QuickSIN.

That consistency was more related to SIN performance than gradience aligns with converging work suggesting response consistency is a stable, trait-like property of behavior and thus more likely to play a role in other perceptual processes.^11,46^ In contrast, perceptual gradiency (as indexed by identification slopes) seems more malleable and may change across different phonetic contrasts and acoustic-phonetic stimuli.^12,14,18^ This questions the generalizability of slope measures as a stable metric of speech categorization abilities (cf. Ref. 43). Instead, consistency may be a more robust index of perception. In our previous work, we measured categorization “listening strategy” *via* identification curve slopes.^8^ Future work should consider analyzing gradiency (slope) and consistency as covarying measures of listening strategy for a more complete picture of perceptual abilities.

Aside from perceptual categorization abilities, cognition is also critical for SIN listening. Prior work has found only a weak relationship between gradiency and WM.^7^ Our results converge with Kapnoula *et al.*,^7^ who found that links between categorization gradience and SIN perception were no longer significant when accounting for listeners' WM, although this study did not report on the relationship between consistency and WM. We similarly found gradience was correlated with WM capacity, although consistency was not. More gradient listeners had better WM spans, suggesting gradience but not consistency may depend on WM capacity, although gradience seems to be generally independent of other cognitive processes like control, attention, or inhibition.^22^ Aligning with Kapnoula *et al.*,^7^ we found categorization slopes did not predict SIN scores on any tasks when accounting for WM capacity. However, WM capacity predicted SIN performance on the HINT, underscoring the well-known advantage of superior WM in SIN comprehension.^23–26^ WM scores were independently correlated with the QuickSIN as expected [Fig. 2(C)], but this correlation did not survive when we account for categorization abilities. The fact that WM predicts HINT performance in our model indicates that the relationship survives accounting for categorization skills, which notably did not predict HINT scores. The lack of significance for other SIN tests does not necessarily contradict the well-documented relationship between WM ability and SIN performance but rather indicates that this relationship dissolves when accounting for other perceptual-cognitive factors.^47^

There are several limitations of the current study. First, is the modest sample. Our sample size was based on targets needed for ongoing electroencephalography studies in our lab and detecting robust category-level effects in the speech-evoked potentials.^8,27,48–50^ As such, the behavioral data herein establish a categorization-SIN relation to examine in future neuroimaging studies. Still, future work should replicate this work in larger samples to determine how generalizable our findings are, including populations with different linguistic background and acoustic-phonetic inventories (cf. Refs. 10, 12, and 51). Second, we used a single vowel continuum to characterize categorization. Although vowels allow for more perceptual ambiguity and gradience than consonant-vowel (CV) continua^2,8^ and are better linked to higher-level linguistic abilities,^16^ it is possible other acoustic-phonetic continua may yield different results. However, we find this unlikely given that listener's degree of perceptual categoricity is highly stable whether labeling vowel or CV continua.^43^ Although categorization consistency is probably stable across continua, gradience may depend on the specific continuum used for assessment^12^ (cf. Ref. 43) Last, it is conceivable listeners perceived a tertiary vowel category between our “u” and “ɑ” end points given the broad categories of vowel space, especially with respect to listener dialect.^52^ However, our RT data suggest otherwise. RTs are a positive function of uncertainty and slow near a phonetic boundary.^5^ Thus, the single peak in the RTs suggests the presence of only a single rather than multiple category boundaries. Still, assessing these relationships across multiple continua and with more continuum steps is necessary to determine the generalizability of our findings.

Collectively, our results suggest that perceptual consistency in speech sound labeling may play an underappreciated role in governing SIN comprehension skills, even in normal hearing adults. WM capacity is often difficult to train and thus might be a limited target for programs designed to improve SIN listening.^53^ Our data imply that improving perceptual consistency during speech listening may offer an easier and potentially more robust transfer to SIN outcomes than attempting to train cognitive factors.

## Data Availability

The data that support the findings of this study are available from the corresponding author upon reasonable request.

## References

[1] A. M. Liberman, F. S. Cooper, D. P. Shankweiler, and M. Studdert-Kennedy, “Perception of the speech code,” Psychol Rev. 74, 431–461 (1967). 10.1037/h0020279 4170865

[2] B. Pisoni, “Auditory and phonetic memory codes in the discrimination of consonants and vowels,” Percept. Psychophys. 13, 253–260 (1973). 10.3758/BF03214136 23226880 PMC3515632

[3] A. M. Liberman, K. S. Harris, H. S. Hoffman, and B. C. Griffith, “The discrimination of speech sounds within and across phoneme boundaries,” J. Exp. Psychol. 54, 358–368 (1957). 10.1037/h0044417 13481283

[4] R. L. Goldstone and A. T. Hendrickson, “Categorical perception,” WIREs Cogn. Sci. 1, 69–78 (2010). 10.1002/wcs.26 26272840

[5] D. B. Pisoni and J. Tash, “reaction times to comparisons within and across phonetic categories,” Percept. Psychophys. 15, 285–290 (1974). 10.3758/BF03213946 23226881 PMC3515635

[6] B. McMurray, R. N. Aslin, M. K. Tanenhaus, M. J. Spivey, and D. Subik, “Gradient sensitivity to within-category variation in words and syllables,” J. Exp. Psychol. Hum. Percept. Perform. 34, 1609–1631 (2008). 10.1037/a0011747 19045996 PMC3011988

[7] E. C. Kapnoula, M. B. Winn, E. J. Kong, J. Edwards, and B. McMurray, “Evaluating the sources and functions of gradiency in phoneme categorization: An individual differences approach,” J. Exp. Psychol. Hum. Percept. Perform. 43, 1594–1611 (2017). 10.1037/xhp0000410 28406683 PMC5561468

[8] R. Rizzi and G. M. Bidelman, “Functional benefits of continuous vs categorical listening strategies on the neural encoding and perception of noise-degraded speech,” Brain Res. 1844, 149166 (2024). 10.1016/j.brainres.2024.149166 39151718 PMC11399885

[9] P. Fuhrmeister and E. B. Myers, “Structural neural correlates of individual differences in categorical perception,” Brain Lang. 215, 104919 (2021). 10.1016/j.bandl.2021.104919 33524740

[10] E. Kutlu, K. Baxelbaum, E. Sorensen, J. Oleson, and B. McMurray, “Linguistic diversity shapes flexible speech perception in school age children,” Sci. Rep. 14, 28825 (2024). 10.1038/s41598-024-80430-1 39572753 PMC11582665

[11] H. Kim, B. McMurray, E. Sorensen, and J. Oleson, “The consistency of categorization-consistency in speech perception,” Psychon. Bull. Rev. 32, 2246–2258 (2025). 10.3758/s13423-025-02700-x 40274721 PMC12426149

[12] E. Myers, M. Phillips, and E. Skoe, “Individual differences in the perception of phonetic category structure predict speech-in-noise performance,” J. Acoust. Soc. Am. 156, 1707–1719 (2024). 10.1121/10.0028583 39269161 PMC11401644

[13] E. Sorensen, J. Oleson, and B. McMurray, “A Bayesian hierarchical model for the analysis of visual analogue scaling tasks,” Stat. Methods Med. Res. 33, 953–965 (2024). 10.1177/09622802241242319 38573790 PMC13242098

[14] E. C. Kapnoula, J. Edwards, and B. McMurray, “Gradient activation of speech categories facilitates listeners' recovery from lexical garden paths, but not perception of speech-in-noise,” J. Exp. Psychol. Hum. Percept. Perform. 47, 578–595 (2021). 10.1037/xhp0000900 33983791 PMC9069052

[15] B. McMurray, “The myth of categorical perception,” J. Acoust. Soc. Am. 152, 3819–3842 (2022). 10.1121/10.0016614 36586868 PMC9803395

[16] H. Kim, J. Klein-Packard, E. Sorensen, and J. Oleson, “Speech categorization consistency is associated with language and reading abilities in school-age children: Implications for language and reading disorders,” Cognition 263, 106194 (2025). 10.1016/j.cognition.2025.106194 40411970 PMC13274561

[17] P. Fuhrmeister, M. C. Phillips, D. B. McCoach, and E. B. Myers, “Relationships between native and non-native speech perception,” J. Exp. Psychol. Learn. Mem. Cogn. 49, 1161–1175 (2023). 10.1037/xlm0001213 36757985

[18] C. T. Honda, M. Clayards, and S. R. Baum, “Exploring individual differences in native phonetic perception and their link to nonnative phonetic perception,” J. Exp. Psychol. Hum. Percept. Perform. 50, 370–394 (2024). 10.1037/xhp0001191 38300566

[19] B. Schouten, E. Gerrits, and A. van Hessen, “The end of categorical perception as we know it,” Speech Commun. 41, 71–80 (2003). 10.1016/S0167-6393(02)00094-8

[20] K. S. Apfelbaum, E. Kutlu, B. McMurray, and E. C. Kapnoula, “Don't force it! Gradient speech categorization calls for continuous categorization tasks,” J. Acoust. Soc. Am. 152, 3728–3745 (2022). 10.1121/10.0015201 36586841 PMC9894657

[21] D. W. Massaro and M. M. Cohen, “Categorical or continuous speech perception: A new test,” Speech Commun. 2, 15–35 (1983). 10.1016/0167-6393(83)90061-4

[22] E. J. Kong and J. Edwards, “Individual differences in categorical perception of speech: Cue weighting and executive function,” J. Phonetics 59, 40–57 (2016). 10.1016/j.wocn.2016.08.006 PMC5423668 28503007

[23] J. Yoo and G. M. Bidelman, “Linguistic, perceptual, and cognitive factors underlying musicians' benefits in noise-degraded speech perception,” Hear. Res. 377, 189–195 (2019). 10.1016/j.heares.2019.03.021 30978607 PMC6511496

[24] A. Dryden, H. A. Allen, H. Henshaw, and A. Heinrich, “The association between cognitive performance and speech-in-noise perception for adult listeners: A systematic literature review and meta-analysis,” Trends Hear. 21, 21 (2017). 10.1177/2331216517744675 PMC5734454 29237334

[25] C. Füllgrabe and S. Rosen, “On the (un)importance of working memory in speech-in-noise processing for listeners with normal hearing thresholds,” Front. Psychol. 7, 1268 (2016). 10.3389/fpsyg.2016.01268 27625615 PMC5003928

[26] D. B. Pisoni and A. E. Geers, “Working memory in deaf children with cochlear implants: Correlations between digit span and measures of spoken language processing,” Ann. Otol. Rhinol. Laryngol. 109, 92–93 (2000). 10.1177/0003489400109S1240 PMC3429114 11141023

[27] J. A. Carter, E. H. Buder, and G. M. Bidelman, “Nonlinear dynamics in auditory cortical activity reveal the neural basis of perceptual warping in speech categorization,” JASA Express Lett. 2, 045201 (2022). 10.1121/10.0009896 35434716 PMC8984957

[28] G. M. Bidelman, S. Moreno, and C. Alain, “Tracing the emergence of categorical speech perception in the human auditory system,” NeuroImage 79, 201–212 (2013). 10.1016/j.neuroimage.2013.04.093 23648960

[29] J. Peirce, J. R. Gray, S. Simpson, M. MacAskill, R. Höchenberger, H. Sogo, E. Kastman, and J. K. Lindeløv, “PsychoPy2: Experiments in behavior made easy,” Behav. Res. 51, 195–203 (2019). 10.3758/s13428-018-01193-y PMC6420413 30734206

[30] M. C. Killion, P. A. Niquette, G. I. Gudmundsen, L. J. Revit, and S. Banerjee, “Development of a quick speech-in-noise test for measuring signal-to-noise ratio loss in normal-hearing and hearing-impaired listeners,” J. Acoust. Soc. Am. 116, 2395–2405 (2004). 10.1121/1.1784440 15532670

[31] M. Nilsson, S. D. Soli, and J. A. Sullivan, “Development of the Hearing In Noise Test for the measurement of speech reception thresholds in quiet and in noise,” J. Acoust. Soc. Am. 95, 1085–1099 (1994). 10.1121/1.408469 8132902

[32] R. H. Wilson, “Development of a speech-in-multitalker-babble paradigm to assess word-recognition performance,” J. Am. Acad. Audiol. 14, 453–470 (2003). 10.1055/s-0040-1715938 14708835

[33] D. Wechsler, *WAIS-IV: Wechsler Adult Intelligence Scale*, 4th ed. (Psychological Corp., San Antonio, TX, 2008).

[34] H. H. Schütt, S. Harmeling, J. H. Macke, and F. A. Wichmann, “Painfree and accurate Bayesian estimation of psychometric functions for (potentially) overdispersed data,” Vision Res. 122, 105–123 (2016). 10.1016/j.visres.2016.02.002 27013261

[35] G. M. Bidelman, L. C. Bush, and L. M. Boudreaux, “Effects of noise on the behavioral and neural categorization of speech,” Front. Neurosci. 14, 14 (2020). 10.3389/fnins.2020.00153 32180700 PMC7057933

[36] D. Bates, M. Mächler, B. Bolker, and S. Walker, “Fitting linear mixed-effects models using lme4,” J. Stat. Soft. 67, 1–48 (2015). 10.18637/jss.v067.i01

[37] A. Parbery-Clark, E. Skoe, and N. Kraus, “Musical experience limits the degradative effects of background noise on the neural processing of sound,” J. Neurosci. 29, 14100–14107 (2009). 10.1523/JNEUROSCI.3256-09.2009 19906958 PMC6665054

[38] S. Gordon-Salant and P. J. Fitzgibbons, “Temporal factors and speech recognition performance in young and elderly listeners,” J. Speech. Lang. Hear. Res. 36, 1276–1285 (1993). 10.1044/jshr.3606.1276 8114494

[39] G. M. Bidelman, M. W. Weiss, S. Moreno, and C. Alain, “Coordinated plasticity in brainstem and auditory cortex contributes to enhanced categorical speech perception in musicians,” Eur. J. Neurosci. 40, 2662–2673 (2014). 10.1111/ejn.12627 24890664

[40] G. M. Bidelman and C. Alain, “Musical training orchestrates coordinated neuroplasticity in auditory brainstem and cortex to counteract age-related declines in categorical vowel perception,” J. Neurosci. 35, 1240 (2015). 10.1523/JNEUROSCI.3292-14.2015 25609638 PMC6605547

[41] G. M. Bidelman, J. W. Villafuerte, S. Moreno, and C. Alain, “Age-related changes in the subcortical–cortical encoding and categorical perception of speech,” Neurobiol. Aging 35, 2526–2540 (2014). 10.1016/j.neurobiolaging.2014.05.006 24908166

[42] D. Lüdecke, D. Makowski, M. S. Ben-Shachar, I. Patil, P. Waggoner, B. M. Wiernik, R. Thériault, V. Arel-Bundock, M. Jullum, E. Bacher, and J. Luchman, “Performance: Assessment of regression models performance,” https://cran.r-project. org/web/packages/performance/index.html.

[43] G. M. Bidelman, F. Bernard, and K. Skubic, “Hearing in categories and speech perception at the ‘cocktail party,’ ” PLoS One 20, e0318600 (2025). 10.1371/journal.pone.0318600 39883695 PMC11781644

[44] J. Krizman, A. R. Bradlow, S. S.-Y. Lam, and N. Kraus, “How bilinguals listen in noise: Linguistic and non-linguistic factors,” Biling. Lang. Cogn. 20, 834–843 (2017). 10.1017/S1366728916000444

[45] R. H. Wilson, R. A. McArdle, and S. L. Smith, “An evaluation of the BKB-SIN, HINT, QuickSIN, and WIN materials on listeners with normal hearing and listeners with hearing loss,” J. Speech. Lang. Hear. Res. 50, 844–856 (2007). 10.1044/1092-4388(2007/059) 17675590

[46] B. W. L. Wong, A. G. Samuel, and E. C. Kapnoula, “Language-specific or universal? The nature and roles of consistency and gradiency in speech perception,” available at https://sciety.org/articles/activity/10.31234/osf.io/t5zbc.

[47] Forward and reverse digit span scores were only marginally correlated, suggesting they assess different aspects of WM. Reverse digit spans require higher cognitive load with mental manipulation of digits for proper recall and forward digit span only requiring simple repetition, aligning with findings that distinct brain regions are associated with each task.

[48] J. A. Carter and G. M. Bidelman, “Perceptual warping exposes categorical representations for speech in human brainstem responses,” NeuroImage 269, 119899 (2023). 10.1016/j.neuroimage.2023.119899 36720437 PMC9992300

[49] J. Alho, B. M. Green, P. J. C. May, M. Sams, H. Tiitinen, J. P. Rauschecker, and I. P. Jääskeläinen, “Early-latency categorical speech sound representations in the left inferior frontal gyrus,” NeuroImage 129, 214–223 (2016). 10.1016/j.neuroimage.2016.01.016 26774614 PMC7380513

[50] G. M. Bidelman, A. York, and C. Pearson, “Neural correlates of phonetic categorization under auditory (phoneme) and visual (grapheme) modalities,” Neuroscience 565, 182–191 (2025). 10.1016/j.neuroscience.2024.11.079 39631659 PMC11700760

[51] G. M. Bidelman and W. L. Chung, “Tone-language speakers show hemispheric specialization and differential cortical processing of contour and interval cues for pitch,” Neuroscience 305, 384–392 (2015). 10.1016/j.neuroscience.2015.08.010 26265549

[52] G. A. Miller and P. E. Nicely, “An analysis of perceptual confusions among some English consonants,” J. Acoust. Soc. Am. 27, 338–352 (1955). 10.1121/1.1907526

[53] A. B. Morrison and J. M. Chein, “Does working memory training work? The promise and challenges of enhancing cognition by training working memory,” Psychon. Bull. Rev. 18, 46–60 (2011). 10.3758/s13423-010-0034-0 21327348

